# Embracing uncertainty: rethinking migration policy through pastoralists’ experiences

**DOI:** 10.1186/s40878-022-00277-1

**Published:** 2022-01-21

**Authors:** Natasha Maru, Michele Nori, Ian Scoones, Greta Semplici, Anna Triandafyllidou

**Affiliations:** 1 Institute of Development Studies (IDS), PASTRES Programme, University of Sussex, Library Rd, Falmer, Brighton, BN1 9RE UK; 2 PASTRES Programme, European University Institute, Via dei Roccettini, 9, 50014 San Domenico di Fiesole (FI), Italy; 3 Institute of Development Studies (IDS), PASTRES Programme, University of Sussex, Library Rd, Falmer, Brighton, BN1 9RE UK; 4 PASTRES Programme, European University Institute, Via dei Roccettini, 9, 50014 San Domenico di Fiesole (FI), Italy; 5 Faculty of Arts, Department of Sociology, Ryerson University, 350 Victoria street, Toronto, M5B 2K3 Canada

**Keywords:** Uncertainty, Pastoralism, Reliability professionals, International migration, Global compact

## Abstract

Today there is a disjuncture between migration flows that are complex, mixed and constantly evolving and the emerging global migration governance paradigm that seeks to impose clarity, certainty, regularity and order. Addressing the gap between policies and realities, this article explores lessons for migration policy and governance from mobile pastoralists’ experience. Using examples from human migration flows within and between Europe and Africa and insights from pastoral systems from India, Italy and Kenya, the article identifies important similarities between international migration and pastoral mobility. We focus on four interconnections: both international migration and pastoral mobility show multi-directional and fragmented patterns; both involve multiple, intersecting socio-economic, political, cultural and environmental drivers; both must respond to non-linear systems, where critical junctures and tipping points undermine clear prediction and forecasts, making social navigation and reliability management more useful concepts than risk-based prediction and control and finally for both uncertainty is not conceived of as a state of crisis but an inherent feature, pregnant with possibility and hope. Building on these four points, and drawing from pastoralists’ experiences, we propose some methodological, practical and policy reflections for bridging the disjuncture between migration realities on the ground and global migration governance policies and discourses.

## Introduction

Migration is a feature of modern life, both across borders and within countries (King, 2012; King & Skeldon, 2010). The 2019 United Nations International Migration Report estimated that there were 272 million international migrants, increasing at a rate of 2.5% a year (United Nations, 2019:3). Add in those who move for work more locally, migration is a lived experience of many across the world. What is seen as uncontrolled, irregular migration, especially as a result of the consequences of conflict and the collapse of economies, is seen as a threat, and in need of management and control. To respond to this, a new global governance framework has been emerging since the mid-2000s culminating with the adoption of the Global Compact for Safe, Orderly and Regular Migration (GCM) and the Global Compact on Refugees (GCR) in 2018 (Ferris & Martin, 2019). The two Compacts though differ in their nature with the former offering a broad, voluntary framework for regulating international migration and providing migrants access to basic services, while the latter is a clearer and narrower document rooted in existing legal commitments (Kainz et al., 2020) While both Compacts remain voluntary and non-binding they clearly offer an important blueprint that shapes regional and global migration consultations.

Such global compacts, which are central to western national framings of migration policy, are focused on risk management, predictability and stability. Despite being at the core of policy debates, they do not necessarilyreflect the realities and lived experiences of migration, as is recognised by a large literature on contemporary migration (e.g., Carling & Collins, 2018; De Haas, 2010; Erdal et al., 2021). As we discuss below, real migration experiences are much more uncertain, dominated by unpredictable events and high levels of variability. Safety is of course sought by migrants, but not necessarily through order and regularity, features that may constrain rather than enable flexibility and adaptive response. The framing of ‘safe, orderly and regular’ migration that dominates particularly the GCM, is one derived from countries who want to manage irregular migration. It is not rooted in the realities of migrations and the understandings of migrants themselves, where conditions of uncertainty dominate.

In this article, we explore the implications of embracing uncertainty in migration policy, drawing from the experiences of those who move regularly, including both mobile pastoralists and migrants. We argue that embracing uncertainty—where we don’t know future outcomes—and offsetting ignorance—where we don’t know what we don’t know—is essential in making the most out of mobility for successful, productivelivelihoods), where ‘reliability’ is generated from highly variable settings (Roe, 2020; Scoones, 2019). By ‘uncertainty’ we mean those conditions where knowledge about the future is unknown, even if the possible outcomes are known (Stirling, 2010). Such a condition of indeterminate knowledge contrasts with a ‘risk’ framing, where the future is assumed to be predictable and calculable and where management and control is the dominant intervention in policy and practice (Scoones & Stirling, 2020). A focus on uncertainty suggests a very different politics, from which follow contrasting approaches to how the variable conditions are seen and responded to in policy. Embracing uncertainties, as well as acknowledging ignorance, in the face of high levels of variability is central to a ‘high reliability management’ approach, widely discussed in relation to ‘critical infrastructures’ (Roe, 2016; Roe & Schulman, 2008). Managers are expected to convert variable inputs into reliable outputs—such as in electricity or water supply systems—and so must be aware of both wider systems dynamics and regular patterns and track between them. Such ‘reliability professionals’ or ‘mess managers’ are seen as crucial to ensuring reliability and the continued supply of system functions and outputs (Roe, 2016). In what follows, we argue that responding to uncertainty and generating reliability are at the heart of successful mobile responses, and central to the practices of both pastoralists and international migrants.

We drawon case material from Africa, Asia and Europe to explore the mobile practices of pastoralists—livestock-keepers who make use of extensive rangelands in ways that very often cross borders—helping to connect debates about international migration with long-standing discussions of mobility in pastoral contexts. Unpredictable, variable conditions dominate pastoral areas, where herders must mobilise an array of responses to confront uncertain conditions (Nori, 2019, 2021; Scoones, 2020), and so must live with and from uncertainty in order to navigate variable rangelands resources, unexpected conflicts, sudden disease outbreak and volatile market conditions (Krätli & Schareika, 2010; Scoones, 1994). This is equally the case among migrants who must deal with border-crossings, difficult transport conditions and variable livelihood opportunities. Yet nevertheless agency, hope and possibility may emerge in the midst of such uncertainties, where fluidity, contingency and unpredictability characterise migration mobilities (De Haas, 2021; Kleist & Thorsen, 2016). The global COVID-19 pandemic has reinforced these dynamics, with new sources of uncertainty added, both for pastoralists (Simula et al., 2020) and for migrants (Chakroborty & Maity, 2020), with questions of im/mobility centre-stage.

Embracing uncertainty and acknowledging ignorance, we argue, has profound implications for how variable conditions are addressed and how reliability, and indeed safety, emerges from complex, messy conditions (Roe, 2016). This is as true for international migrants as it is for pastoralists. These parallels we argue can offer important insights for rethinking migration policy, opening up new foci for dialogue. This has major implications for the research methodologies we use; for thinking about organisations and professional practice and for the design of global governance and policy approaches. Mobility in different shapes and forms and across many scales, whether of animals or people, is central to modern life. In an increasingly mobile world (Bauman, 2007; Cresswell, 2006; Urry, 2000), where flows of finance, commodities, information and people characterise networked global relations (Castells, 2011) and spaces for (selective) mobility are opened up, we can learn from pastoralists’ experiences in reframing global migration policy frameworks in ways that embrace uncertainty. Throughout the article, we argue that centring uncertainty in the migration policy debate opens up new horizons for policy dialogue about the future of migration more generally. Such a reflection becomes all the more timely at a time of a persisting and intense global uncertainty arising from the pandemic emergency (Triandafyllidou, 2022).

## Framing migration, mobility and uncertainty

Migration has been intensifying and diversifying in the last 30 years, accelerating in recent times due to major conflicts and regional political and economic shifts (McAuliffe & Triandafyllidou, 2021,2021). Migration realities have become increasingly dynamic, and this is reflected in the scientific vocabulary used to study migration. We used to organise our thinking in dichotomous ways, with reference to ‘origin’ and ‘destination’ countries, and ‘push’ and ‘pull’ factors. In the last decade, however, we increasingly study transit countries, fragmented migration journeys, focusing on aspirations, capabilities, needs and experiences (Carling & Collins, 2018; De Haas, 2010, 2021; Triandafyllidou, 2021). This shift highlights the importance of uncertainty, but the lessons have yet to find their way into mainstream policy frameworks; although as we discuss further below much migration research is well-aware of such dynamics (e.g., Bijak & Czaika, 2020), and so echoes much of the experience that we document below from pastoralist settings.

This section therefore reviews four important challenges to the standard policy framings of international migration today. These challenges are widely discussed in the academic literature on migration, but remain largely ignored by policy frameworks and the ‘safe orderly and regular’ migration rhetoric. These all point to the need for an approach to migration governance that takes uncertainty seriously.

First, the distinction, so often repeated in policy discussions, between countries that are mainly hosts of migrants and those of origin does not hold. Migration is not just about starting and end-points, framed by the idea of a nation state, but involves multi-directional, networked flows (Kalir, 2013). Many countries are sites of emigration, immigration as well as transit or periodic migration. Italy, for example, experiences important out-migration particularly of highly-skilled people, but also significant immigration from both EU and non-EU countries (Colombo & Dalla Zuanna, 2019). Portugal is also an interesting example due to the multi-directional migration networks with other Lusophone countries, notably Brazil or Angola, while emigration to northern European countries continues (Pereira & Azevedo, 2019). A closer look at West African countries such as Senegal, Ghana or Nigeria, considered in Europe as important countries of migrant origin, shows that they are implicated in complex flows of mobility within the wider West African region and towards Europe (Omobowale et al., 2019). Thus, what defines the role of each country is not only the numbers, but its role in the migration process as predominantly an origin or destination country and thus while both Italy and Nigeria may face emigration, immigration or transit migration, Italy’s geopolitical role in international migration governance is that of a destination country, while Nigeria’s is that of an origin country. We need therefore to do away with the notion of ‘origin’ and ‘destination’, or ‘sending’ and ‘receiving’ countries and the linear, sedentary biases that they imply (Bakewell, 2008), and rather uncover the power relations and contextual features that lie behind such designations.

Second, decisions to migrate, stay, return or move on are taken in a dynamic environment and are shaped by multiple drivers (Belloni, 2016a, 2016b; Koikkalainen et al,*.* 2016; Bal, 2014), not simple push and pull factors as is often assumed. While there has been increased emphasis on mixed migration flows, notably on asylum seekers and economic migrants travelling the same routes, it is only recently that there is an acknowledgement that the distinction between forced and voluntary migration is a matter of degree (cf. Jubilut 2019). Recent research has highlighted a range of strategies as central to migrant practices. These include applying for asylum to obtain a temporary legal status (Bloch et al., 2011); going underground when other options are not available (Reyneri, 1998); drawing on ethnic networks (Moret, 2016); responding creatively to tighter border controls by passing as tourists (Chavez, 2011); or using knowledge of other migrants’ past experience or international law to insist on coastguard services to rescue them (Mainwaring, 2016). Contrary to policies that seek to create sharp distinctions between economic migration and asylum-seeking or legal and irregular migration, acting to control and regularise assumed illegal flows, such distinctions are blurred (Spencer & Triandafyllidou, 2020). This means exploring how migrants exercise their agency in ways that go beyond the contrast between legal, stable and orderly versus illegal, volatile and irregular distinction that dominates global migration policy (Triandafyllidou, 2017). This does not mean ignoring the degrees of constraint that some migrants face but rather to put migration into the context of wider processes of socio-economic and political transformation (De Haas, 2021).

Third, the media, policy and political discourses on human mobility portray both migration and asylum seeking as a crisis, or as the result of a crisis—whether economic, environmental or political. A focus on crises however may unhelpfully reinforce a sharp distinction between humanitarian and economic migration flows, and the assumption that there is a stable equilibrium to which the system will return following interventions to improve stability, regularity and ‘resilience’. Despite a ‘crisis’ event, many by necessity must continue living daily lives, in the face of what is seen externally as a sudden, catastrophic disaster, but which is locally construed as part of a permanent condition of uncertainty. A focus on events, linked to crises, provokes efforts to create forecasts in terms of the future size and direction of migration flows—for example, this is what the European Border Agency, Frontex, seeks to achieve with its Risk Analysis quarterly and annual reports (Frontex, 2020). However, forecasted predictions have repeatedly been proven wrong (Bahna, 2008). In complex, tele-connected systems operating across scales, such shifts in system dynamics may be highly uncertain and very far from predictable (Liu et al., 2013), influenced by critical junctures and tipping points. Given this complexity and the uncertainties of migration processes, the notion of social navigation (Vigh, 2009) can be helpful in conceptualising the different forms of agency that migrants develop (Triandafyllidou, 2019). Migration is a multi-dimensional (social, spatial, temporal) process that develops in non-linear ways, with several transit phases and places that can also involve moments of being ‘suspended’, both physically (because ‘stuck’ in a place) and legally (because having an ‘irregular’ status) (Oelgemoeller, 2011). In other words, it is a fragmented, highly contingent and unpredictable journey that can involve a varying ‘pace’, slowing down, speeding up or pausing. Migration includes an important aspect of improvisation (Brigden, 2016), which is both territorial (in terms of changing routes) and social (in terms of roles and identities that migrants on the move perform, defying national borders).

Fourth, in popular and policy discourses, migration is often framed as risky, challenging, dangerous and violent (Krzyzanowski et al., 2018). But movement can also be understood as embodying flexibility, autonomy, aspiration, desire, expectation and imagination (Belloni, 2016a, 2016b; Thorsen, 2020). Uncertainty thus may be seen as a state of anxiety and doom, emerging from potential violence and risk – as it is assumed to be in Western ways of thinking and as is codified in international organisations’ views of irregular migration – or alternatively, as a focus of potentiality and a source of hope. In the accounts of young migrants from sub-Saharan Africa (Bachelet, 2016; Thorsen, 2020), uncertainty is pregnant with hope and possibility, becoming a lever for action. This interpretation is framed in cultural and religious conceptions of life, aspiration and destiny: it is about being in the hands of God, sometimes framed in a narrative of adventure (Thorsen, 2020: 145–146), passing time and becoming an adult (Jeffrey, 2010) and of chance and gambling (Belloni, 2016b).

There is thus a fundamental disconnect between what field-based research has shown on the dynamic, uncertain and complex set of drivers and human agency that shape migration practices and the framing that dominates policy-making and global governance of migration. Instead of aiming to predict, manage, regulate and control, there is a clear need to admit non-linear, open-ended, multi-faceted, uncertain possibilities for migration and the impossibility of control.

In the rest of the article, we draw on insights from the world of pastoralists, based on on-going fieldwork in India, Italy and Kenya, and argue for an alternative perspective on mobility that accepts uncertainty and unpredictability. When uncertainty is embraced and the mirage of linear order, equilibrium and control is abandoned, we argue that more effective governance approaches and institutions can emerge that manage the ‘mess’ of migration, rather than seeking to predict and manage through imposing control and order, although frequently failing to do so.

## Learning from pastoralists’ experiences: implications for rethinking migration framings

In this section, we reflect on the four themes emerging from debates about international migration discussed above in relation to pastoralists’ experiences. Pastoralists face a range of uncertainties not just generated through variabilities in their biophysical environment (Behnke et al., 1993), but increasingly from economic, social and political relations (Semplici, 2020a; Scoones, 2020). From pastoralists’ perspectives, unlike those of the policy-maker, uncertainty characterises all facets of life. Milk yields follow volatile rainfall patterns; livestock prices respond to global value chains; food may be available in the market or be offered as part of government distributions; development programmes may happen, be delayed or fail. In response, pastoralists deploy a range of flexible, adaptive and proactive strategies to exploit this variability. They harness uncertainty in order to receive greater returns than if the environment was more stable (Krätli, 2019). In so doing, movement can be seen as a networked flow, underpinned by a range of skilled practices that enhance reliability in the face of intersecting uncertainties.

### Beyond source and destination framings – mobility as a flow

Pastoralists must engage dynamically with uncertainty and variability, and as a result they challenge linear, uniform and predictable notions of mobility. Rarely do they move predictably from point A to B; a move from dry to wet areas or from home to host territories. Moving and stopping is part of a continuous and contingent flow, where ideas of mobility and immobility are not opposites, but part of the same experience (Maru, 2020). The lived experiences of mobility rupture the binaries of start and stop, source and destination, fixed and flexible, mobility and immobility as pastoralists seek to respond opportunistically to contextual dynamics.

Just as with international migration, the rhythms and routines of pastoral mobility may involve multiple stops and pauses, loops and flows and perhaps no fixed destination. Movements include overlapping and nested circuits of long-distance and short-distance movements, annual, seasonal and daily movements, regularised and spontaneous movements, restricted and unrestricted movements. For example, Rabari pastoralists in Kachchh in Gujarat, India, are embedded in various overlapping circuits of mobility. They remain in migrating camps throughout the year participating in an annual migration cycle, which seasonally moves from the commons to cotton-growing areas and then to wheat-growing areas. They stop along the way at temples during their migration route, fulfilling both livelihood based and religious obligations. At the micro-level, the animals move around the camp every day, while the camp itself moves every few days with the occasional movement of members away from the camp to, perhaps, religious sites or even back to the village (Maru, 2020).

As with the Rabari, among the Turkana in northern Kenya decision-making on the direction and speed of movement often occurs on the go. This requires skills and aptitudes for negotiating complex movements—whether to far-flung patches of grazing in the dry season or to safe places to avoid raids and conflict. In seeking new grazing, water, labour sources or market and trade opportunities, pastoralists must scout out what is available, where, under what conditions and with what potential risks. The same applies when threats are on the horizon, whether an animal disease identified in a neighbouring district or a swarm of locusts affecting a certain area of valued grassland. These knowledge-brokering and real-time management practices involve drawing on social networks—among kin, clan and other relationships—making use of the ubiquitous mobile phone, and if not, sending out messengers on motorbikes into the rangelands to garner information. Pastoralists therefore must both continuously scan the horizon for future threats and understand the realities on the ground in real-time in order to manage mobilities (Tasker & Scoones, 2022; cf. Roe et al., 1998).

Practices of mobility are thus embedded in networks, with knowledgeable and well-connected herders at the centre. There may be key people at certain nodes, such as milk collectors or market traders, very often acting in concert (de Bruijn et al., 2016; Nori, 2021). While they may draw on other types of information, such as from government extension services, satellite monitoring bulletins or market information systems, these are not the primary sources influencing movement choices. They triangulate external information with their grounded knowledge and local experience in real time. Reliability and safety thus emerges through combining diverse sources of knowledge across networks and relaying this so that others are able to respond flexibly and adaptively, resulting in uncertainties being managed and sources of ignorance, where real dangers lie, being avoided (Roe, 2016). Responses must be continuous, and information is updated as uncertain conditions unfold. Unlike formalised Early Warning Systems that are designed to spot crises, providing prescriptions on how to control variability, pastoralists must instead embrace uncertainty (Caravani et al., 2021; Krätli et al., 2013).

### Intersecting drivers of mobility

Beyond the search for pasture and water, pastoral mobility is a response to multiple drivers. The boundaries between push and pull factors, choice and constraints, aspirations and capacities are all blurred. Pastoral mobilities may be influenced by several considerations simultaneously; accessing certain fodder resources at certain times; responding to economic incentives and market opportunities; or redefining priorities in relation to policy-based subsidies. Local economies and options for movement may be affected too by wider networks linked to trade or remittance flows.

In Sardinia in Italy, for example, the pastoral economy depends on the Pecorino Romano cheese global value chain, and so must be highly responsive to shifts in prices of sheep milk and fodder, as well as the form and amount of subsidies from the state or European Union. Mediterranean pastoralism, including in Sardinia, has undergone major changes over the past decades. Mobility patterns have altered due to structural shifts in rural economies, encouraging in-migration into remote, mountainous areas that had earlier suffered a long period of depopulation. The Italian government, for example, supported a major migration of pastoralists from the island of Sardinia to the mainland. This migration started as an informal migratory flow, as pastoral families moved to Italy with their animals in search of pastrures and markets, while also fleeing local feuds. A barrage of policy incentives, credit systems and institutional arrangements were later established to encourage further migration and so a new form of pastoralism in mainland Italy (Nori, 2021). However, pastoral mobilities must also respond more contingently, arising from particular circumstances and evolving uncertainties. Whether it makes sense to move animals between mountain/hill and plain pastures or intensify production in the lowlands will depend on how these drivers play out (Nori & Farinella, 2020).

Extreme weather events, animal disease, wildlife attacks and conflict conditions may trigger sudden movements, while large land investment schemes and enclosures may lead to changes to migration paths. New routes of animal movement must be assembled and reassembled through complex negotiations, as is shown in the case of northern Kenya where new investments in commercial agriculture, conservation, infrastructure developments and extractive industries have affected the pastoral landscape (Lind et al. 2020). When movements are not possible, or constrained by conflict for example, in order to sustain livelihoods and keep animals healthy, Kenyan pastoralists adopt new tactics: instead of moving animals they may move fodder, importing hay from other areas, now easily connected by improved roads and mobile phone networks. As a result, new mobilities emerge due to complex drivers resulting in the management of resources in new ways.

Pastoral mobility practices in any setting are always highly differentiated. Changes in movement patterns among pastoralists may emerge from long-term structural changes in economies, as well as policy-induced shifts, all playing out alongside individualised, very particular choices. Movement is rarely completely free, as access to resources is invariably constrained by, for example, conflict between ethnic groups, enclosures due to private investments or the expansion of urban settlements. However, there is an important political distinction between negotiating such constraints voluntarily and forced displacements or resettlement resulting from external policies. In pastoral areas, involuntary, forced displacement is increasingly evident, as the expansion of investment into dryland areas continues, whether through large-scale agriculture, dam projects or renewable energy investments (Lind et al. 2020). Although patterns of forced and voluntary movement are blurred, certain interventions may undermine the flexible adaptability at the core of generating reliability in the face of high levels of variability. Although risk assessments and risk management strategies are applied to displacement and resettlement projects, they rarely appreciate the contexts within which pastoral livelihoods are generated (Rogers, 2020). In such contexts of forced displacement, generating reliability in the face of uncertainties may be impossible as options within the pastoralists’ repertoire are eliminated in settlement schemes or through forced eviction and displacement (Schrepfer and Caterina 2014). In pastoral settings, as with migration more generally, multiple intersecting drivers therefore combine with varying political implications under conditions of high variability and uncertainty, and any simple assessment of predictable movement (or stasis) is quickly upset.

### Navigating uncertainties: rejecting the ‘coping with crisis’ narrative

As in discourses on international migration, mainstream views about pastoralism are still imbued by a language of coping with a harsh environment and responding to ‘crisis’. Yet pastoral mobility is far from a simple coping mechanism: it involves managing grazing itineraries in a way that maximises the use of rangeland resources to secure livelihoods. Mobility enables socio-spatial and socio-economic connectivity that allows pastoralists to navigate contingencies, change and variability. Pastoral mobility is thus a deliberate and carefully considered choice to embrace systemic uncertainties, rather than a momentary decision based on a singular, crisis event.

Indeed, an analysis of the everyday lives and practices of mobility among Turkana herders in northern Kenya has shown that mobility is part of the way people organise their lives (Semplici, 2020a), and not a “temporary response to declining entitlements” (Davies, 1993:60); rather mobility is essential to seize “fields of opportunities” (Goodhand, 2014:19, quoted in Hammond, 2019). Their moving is a response to positive environmental change (land flourishing, increased precipitation, peaking nutritive value in grass), more than to a deterioration of local resources. Movement increases during the the rainy season; while during the dry season people reunite and concentrate around the fewer available resources and wait for the rain to bring change.

Similarly, pastoralists from the dryland region of Kachchh in western India graze in the commons in the monsoon months before moving to mainland Gujarat to graze on crop residues of farmers’ fields in the summer and winter months. They time their migration to match harvests in agricultural hotspots, slowing down and speeding up based on the weather and cropping cycle each year. By doing so they are able to procure fresh and nutritious fodder for their animals throughout the year, and so improving animal health and reproduction. Moreover, they are able to supplement their income in agricultural areas through manure exchange (Maru, 2020). Mobility for pastoralists thus can be seen as a strategy to ensure reliable outputs from a variable supply of inputs such as, for example, continuous milk production from uncertain rainfall and supplies of fodder (Roe, 2020).

Therefore, the ‘coping with crisis’ narrative fails to explain the complexity that shapes pastoral mobilities in the face of rapid economic and environmental change and wider structural constraints. The task of policy frameworks, and the motivation for this article, is to address these constraints in a way that acknowledges the multiple dimensions of uncertainty and accommodates and promotes the strategies of mobile peoples to respond to such uncertainties.

### From danger and risk to possibility and hope

The uncertainties that come with and prompt mobility are not just sources of risk and danger; just as with international migrants, there are other framings centred on possibility and hope that pastoralists articulate. Of course, movements are experienced by different people in different ways. A young, male migrant herder from Romania may experience a great sense of liberation in moving to Italy to herd sheep, connecting with his skills and passions and escaping the confines of family life, deepening poverty and limited individual opportunity. Similarly, the days of crossing over from Kachchh to Gujarat for their annual migration are extremely exciting for pastoralists, but also difficult for women as they must walk long distances and frequent camp changes means more work for them. In Turkana in an encampment along the Urgand escarpment, the days preceding the migration are also filled with excitement. Dances and songs are performed, liquor is brewed in the huts as a sense of trepidation increases around the imminent movement.

Even when physical movement of livestock declines, pastoralists often also have a sense of that they still have a sense of ‘imaginative’ mobility, where they are transported in their minds rather than bodies. Among pastoralists in Turkana, animal diseases are often associated with immobility, standing still and the impossibility to walk is read as the sign of threatening diseases. If immobility signifies illness, mobility is metaphorically conceived as the cure. Treatments involve cutting the affected animal, letting evil spirits holding the animal still go away, thus freeing it from its status of immobility (Semplici, 2020a).

Different spatio-temporal contexts along the journey elicit a range of feelings. Just as for international migrants, movement is very much part of pastoralists’ differentiated identities, and their identifications with diverse places and people, with the experiences and articulations of mobilities differing by age, sex, caste and class. Across such differentiated experiences of mobility, overlapping concepts of time come into play. This is not the ordered temporality of the calendar and clock, but one that is more malleable, able to respond to changing conditions. This sense of multiple flows of time – long and short term – intersect in a culturally-embedded response that is simply not amenable to standard, ordered planning frameworks (cf. Bear, 2016). The idea of ‘orderly’ and ‘regular’ migration, as defined by global policy frameworks for international migration, is at odds with a more flexible, adaptable, responsive modality seen amongst pastoralists, which is much more attuned to uncertainty (Scoones & Nori, 2020). Mobilities, experienced in different ways are not just a response to risk, threat or danger, but open up possibilities and opportunities, reflected in a sense of hope, excitement and freedom. Mobility for pastoralists is therefore not just about coping with crises, but making productive use of uncertainties, exploiting variability to assure livelihoods in a challenging setting (Krätli & Schareika, 2010).

## Implications for migration policy

What are often cast as unruly, backward forms of pastoral mobility that resist sedentarisation and fixed, regularised plans and ways of life are in fact highly effective responses to uncertainty. They are differentiated across social groups and between places, but one of the core principles of pastoralism is that of sustaining mobility, even if the specific practices change. Attempts to limit, manage or control mobility inevitably fail, as such efforts can fundamentally undermine livelihoods. Thus, approaches to pastoral sedentarisation may be appreciated in part because of opportunities for education, health care and jobs in settlements, but to sustain pastoralism, people must split families and continue mobile herding separately (Ahearn & Bumchir, 2016). It is never a complete transition to what is assumed to be the ideal sedentary life (Semplici, 2020b).

For pastoralists, responding to the unpredictability of variable rangeland, market and political settings, requires a range of practices, with flexible mobility at the centre. For migrants, it is similar informal, irregular, often hybrid arrangements allowing flexible movement that are crucial. For both pastoralists and migrants the rigid, formalised rights, rules and regulations of standardised development plans or policy compacts, despite good intentions, often constrain more than they enable. In this article, we have argued therefore that pastoralists – together with insights from the real lives of human migrants—can offer many important insights, and an uncertaintyperspective may hold important implications when thinking about international migration policy and challenging the framing of ‘safe, regular and orderly’ migration that dominates global policy frameworks and the governance of international migration today.

In this section, we highlight three implications for international migration policy as currently framed in mainstream discussions emerging from our analysis, each of which put uncertainty centre-stage: the methodological implications of engaging with uncertainty in mobile settings; the implications for embedding new types of professionalism and associated practices that generate reliability in the face of uncertainty; and the implications for global governance frameworks themselves of taking uncertainty seriously.

### Methodological implications

Methodologies that inform policy, both in the contexts of pastoralism and international migration, may ignore uncertainty and complexity. They count numbers between origin and destination, assess net movements in relation to targets, and evaluate the factors that push and pull in particular defined directions across borders. The requirements of the audit culture, especially as migration has risen up the political agenda as something to be ‘controlled’, require simple measures, risk-based predictions, easily-read dashboards and statistics that show success according to specified metrics (Power, 1997). All these audit approaches ascribe to the desire to measure order and control. They emerge through the effects of a particular style of governmentality seen in full view in the Global Compacts (cf. Barry, 2006). Most crucially, such approaches are unable to apprehend the complexity, uncertainty, ambiguity and contingency of actual migration practice.

A closer look at both pastoral mobility and human migration reveals insights that help improve our methodological approaches, rupturing standard framings. However, although there is a rising consensus about how uncertainty and complexity shapes society today, the ‘methodological infrastructure’ -our standard analytical tools and practices—lags behind. This creates a gap, resulting in inappropriate policy recommendations and frameworks. What then are the methodological challenges encountered in contexts of uncertainty, complexity and ‘mess’? New methodological practices that accept non-linearity, operate in real-time and encourage adaptation are often incompatible with the legacy of standard systems that structure institutional responses (Bowker & Star, 2000). Policy organisations dealing with migration, just as with pastoralism, find it challenging to get out of the box of assuming equilibrium, striving towards stability and pushing predictions and plans. Data collection is frequently sequential and ordered, focused on gaining clear numbers, rather than opening up to serendipity and flexible, adaptive learning. Indicators that emerge focus on averages rather than ranges, and often hide variability and heterogeneity. When data are analysed, categories are used—such as sources, destinations, routes and corridors—that hide ambiguity and impose a particular frame. More contingent, variable practices such as emic, experiential, affective and embodied knowledges that are so central to mobility are thus hidden from view. Recommendations that then follow are reinforced by a particular drive to regulate and control. Knowledge is therefore co-constructed with particular, powerful, institutionalised political and social orders, and a consensus is maintained (Jasanoff, 2004).

Research on pastoralism has begun to challenge this conventional methodological infrastructure (Krätli, 2019; Krätli et al., 2016; Pappagallo & Semplici, 2020), drawing on diverse insights from different methodological traditions (Law, 2004). Notions of stability in pastoral systems have been long challenged by ecologists who identified the non-equilibrium characteristics of dryland ecosystems (Ellis & Swift, 1988). This had major implications for how management and policy response was conceived (Scoones, 1994), with broader implications for social inquiry (Scoones, 1999). However, acknowledging variability—and with this uncertainty, contingency, ambiguity, even ignorance—makes research more challenging, and old patterns are easily resorted to even in pastoral settings (Krätli, 2019). Understanding systems that are undergoing continuous, unpredictable change equally makes standard modelling approaches inadequate, while forecasting and prediction are impossible. In the same way, a standard survey may offer a meaningless snapshot. Alternatives centred on a longer-term process of engaged monitoring and learning, across different spatio-temporal scales, offers different insights. These methodological reflections from studies of pastoralism are echoed in qualitative studies of human migration, where agency, contingency, experience and multi-dimensional and intersecting drivers are emphasised as central to effective methodologies for exploring connections between migration and development (e.g., Czaika & Godin, 2021; Erdal et al., 2021).

Policy-makers often require a simple narrative of problem and solution for implementing policy (Roe, 1991), and so become blind to variability and uncertainty in the usually futile search for regularity and control. Variability is therefore seen as something to be managed and uncertainty something to be offset, rather than both being seen as central to system dynamics (Krätli et al., 2016). Thus, the challenge for governance in contexts of uncertainty and mess is a political issue as much as it is a methodological one. Unsettling research practices still anchored to equilibrium views of the world becomes a central challenge for migration research, as it has for work on pastoralism. The result must be making research methods and policy advice messy too (Pappagallo & Semplici, 2020).

### Organisational, professional and practical implications

If predictions are inadequate and standard planning frames do not work, how can reliability in the face of high levels of variability and so uncertainty be realised? Reliability—assuring the stable supply of desired services from a system—emerges when processes allow the transformation of high-input variance into low-output variance. The result may be safety for migrants, or reliable production from livestock for pastoralists. As we have learned, this is not necessarily brought about through regularity and orderliness, as assumed by policy frameworks. Instead, drawing from work on ‘critical infrastructures’ (Roe & Schulman, 2008), we can identify the importance of ‘reliability professionals’ who are able to both scan the horizons for future threats and understand the realities on the ground in real-time. They are the operators in energy supply system control rooms for example, but they also exist in both migrant and pastoral settings (Roe, 2016, 2020).

Reliability thus emerges through combining diverse sources of knowledge across networks and relaying this in real-time so that others are able to respond flexibly and adaptively, resulting in uncertainties being managed and sources of ignorance, where real dangers lie, being avoided. Reliability professionals have a range of skills. They make use of data and are able to deploy heuristic models to make sense of multiple sources of information, but they must also act as brokers, mediators and negotiators within networks, communicating freely and being open and transparent about where uncertainties remain. They must rely on experience, tacit knowledge, diverse senses, emotional intelligence, intuition and awareness. They must also be good network builders, communicators, leaders and negotiators (Roe & Schulman, 2008). While they may not have a formal job description, they are crucial in any infrastructure aiming to deliver services reliably (Roe, 2020). They look very different to the hierarchical management systems and monitoring and evaluation professionals in conventional control-oriented organisations that dominate migration and pastoral development policy.

As our analysis has shown, pastoralists and migrants use the same skills as reliability professionals in critical infrastructures. They draw on networks, deploy technologies and respond in real-time in the search for safe passage and mobility that generates successful livestock production. Standardised plans, regularised systems and orderly processes may not facilitate this, and indeed may do the opposite, undermining flexible, adaptive practices. In this way, uncertainty therefore has to be seen as an opportunity, not something to be controlled or eliminated.

### Implications for global governance frameworks and policy

Embracing uncertainty has a number of important implications for the global governance of migration and the frameworks that guide it. As we have discussed, there is a disconnect between migrants’ practices on the ground and the standardised approaches to the global governance of migration, as epitomised by the Global Compacts.

This is not to say that global governance has no role, as a wider framework for flexible, responsive action to generate reliability will always be necessary, especially when migrations cross borders and international collaboration is required. However, the nation-state framing that is so alien to pastoralists and migrants, but so central to such policies, has to be challenged. Frontiers, just as borders and fences, are conceived and established to protect what is inside, and such ways of organising access contrasts with systems and people that make use of the opportunities afforded by crossing borders as a central pillar of their livelihoods. For pastoralists, this may be for livestock grazing, trade, labour markets or just seeking alternatives to conflict and poverty (Butt, 2016; Regassa et al., 2019; Roba et al., 2018). Indeed, in places where movement is common, national borders may cut across long-existing movement routes, dividing networks and even families. As modern, colonial inventions, nation states that guide policy framings may have less relevance for mobile peoples, where alternative associations and forms of ‘citizenship’ may exist (cf. Horst, 2008; Markakis, 2021).

Whether national or regional policy frameworks governing international migration or the regional policy frameworks defining pastoral policy, such as emerging in Africa from the African Union, IGAD or ECOWAS^1^ (Davies et al., 2018), there is a clear need for a more flexible, plural, decentred approach, premised on a multi-level governance framework that can encompass decentralised local knowledges and practices (Triandafyllidou, 2020), requiring policy to see the world like migrants or pastoralists (Catley et al., 2013). This means a rethinking of ‘global compacts’ premised on regularised stability and order, challenging current framings so asto allow for the enhancement of reliability practices and professionals, putting uncertainty at the centre. This means accepting that addressing international migration or pastoral development necessarily requires working in highly variable settings, and so attempts at calculative prediction and linear control for stability must be abandoned, and with this a new politics of uncertainty, responsibility and accountability developed (Scoones & Stirling, 2020).

As a result, guiding principles of orderliness and regularity need to be replaced by principles of variability, uncertainty and reliability, while safety needs to emerge from people’s own experiences, negotiated in a setting that enables rather than constrains. Metrics that evaluate policy success must, in turn, shift from measuring net movements or framings in terms of defined sources and destination sites to processes of continuous learning and reflection on how movements actually happen, with indicators of success defined by the experience of those who move. The scope and meaning of mobility as a result has to be reframed too, avoiding the deep misunderstandings projected by sedentary perspectives, where movement is controlled in particular spaces, at particular times and for particular purposes in ways that undermine the expertise and livelihoods of pastoralists and migrants alike.

## Conclusion

A pastoralist perspective on migration policy therefore highlights variability, uncertainty and the search for reliability, and so necessitates rethinking expertise, opening to diverse, local knowledges and a different approach to governance that is more flexible, creative and adaptive. This means focusing on those professionals and networks who are able to transform high variability and so uncertain conditions to more stable, predictable outcomes, not through top-down regulation and policy, but through adaptive practice, flexible learning and embedded social relations. This in turn has consequences for the political and accountability relations at the heart of policy, and so what forms of governance arrangement make sense.

As the Turkish author Elif Shafak has explained, different people occupy either liquid or solid lands.^2^ Those making policy, defining development frameworks and constructing global compacts usually occupy the solid lands, where control, stability and management are imagined. This is the minority experience, as most of the world occupies the liquid lands, where uncertainty, mess, contingency and ambiguity dominate. Navigating across the liquid lands requires very different skills and a radically different outlook. This is where understandings from pastoralists, together with the lived experiences of international migrants, as perhaps some of the most experienced practitioners of living with and from uncertainty, become important.

That these diverse experiences of mobility converge is important, suggesting the opportunity for exchange, dialogue and mutual learning. If the majority, with the deep experience of living in the liquid lands, can challenge the minority from the solid lands, confronting the inheritances of colonialism and the narrow framings of progress, modernity and development, then the opportunities for a wider view of migration as productive, hopeful possibility that can embrace uncertainty, opens up. With this, we argue, emerges a fundamental rethinking of the way we frame and implement global policy frameworks and compacts on migration.

## Data Availability

This paper is a meta-analysis and does not include any primary data.

## Footnotes

1 The Intergovernmental Authority on Development (IGAD, https://igad.int/) and the Economic Community of West African States (ECOWAS, https://www.ecowas.int/).

2 https://www.bbc.co.uk/programmes/p092lxzb (final segment).
